# Phosphodiesterase Type 5 Inhibitors Increase Herceptin Transport and Treatment Efficacy in Mouse Metastatic Brain Tumor Models

**DOI:** 10.1371/journal.pone.0010108

**Published:** 2010-04-19

**Authors:** Jinwei Hu, Julia Y. Ljubimova, Satoshi Inoue, Bindu Konda, Rameshwar Patil, Hui Ding, Andres Espinoza, Kolja A. Wawrowsky, Chirag Patil, Alexander V. Ljubimov, Keith L. Black

**Affiliations:** 1 Department of Neurosurgery, Maxine Dunitz Neurosurgical Institute, Cedars-Sinai Medical Center, Los Angeles, California, United States of America; 2 Department of Medicine, Cedars-Sinai Medical Center, Los Angeles, California, United States of America; 3 Department of Surgery, Cedars-Sinai Medical Center, Los Angeles, California, United States of America; 4 David Geffen School of Medicine at University of California Los Angeles, Los Angeles, California, United States of America; Johns Hopkins University, United States of America

## Abstract

**Background:**

Chemotherapeutic drugs and newly developed therapeutic monoclonal antibodies are adequately delivered to most solid and systemic tumors. However, drug delivery into primary brain tumors and metastases is impeded by the blood-brain tumor barrier (BTB), significantly limiting drug use in brain cancer treatment.

**Methodology/Principal Findings:**

We examined the effect of phosphodiesterase 5 (PDE5) inhibitors in nude mice on drug delivery to intracranially implanted human lung and breast tumors as the most common primary tumors forming brain metastases, and studied underlying mechanisms of drug transport. *In vitro* assays demonstrated that PDE5 inhibitors enhanced the uptake of [^14^C]dextran and trastuzumab (Herceptin®, a humanized monoclonal antibody against HER2/*neu*) by cultured mouse brain endothelial cells (MBEC). The mechanism of drug delivery was examined using inhibitors for caveolae-mediated endocytosis, macropinocytosis and coated pit/clathrin endocytosis. Inhibitor analysis strongly implicated caveolae and macropinocytosis endocytic pathways involvement in the PDE5 inhibitor-enhanced Herceptin uptake by MBEC. Oral administration of PDE5 inhibitor, vardenafil, to mice with HER2-positive intracranial lung tumors led to an increased tumor permeability to high molecular weight [^14^C]dextran (2.6-fold increase) and to Herceptin (2-fold increase). Survival time of intracranial lung cancer-bearing mice treated with Herceptin in combination with vardenafil was significantly increased as compared to the untreated, vardenafil- or Herceptin-treated mice (p<0.01). Log-rank survival analysis of mice bearing HER2-positive intracranial breast tumor also showed a significant survival increase (p<0.02) in the group treated with Herceptin plus vardenafil as compared to other groups. However, vardenafil did not exert any beneficial effect on survival of mice bearing intracranial breast tumor with low HER2 expression and co-treated with Herceptin (p>0.05).

**Conclusions/Significance:**

These findings suggest that PDE5 inhibitors may effectively modulate BTB permeability, and enhance delivery and therapeutic efficacy of monoclonal antibodies in hard-to-treat brain metastases from different primary tumors that had metastasized to the brain.

## Introduction

Metastases of various tumors to the brain account for the majority of brain cancers [1]–[3]. The prognosis for patients with brain metastases remains poor with median survival of 4 to 5 months. Effective treatment of brain cancers is in part impeded by a limited transport of anti-tumor therapeutics into the tumors across brain tumor capillaries [4]–[6]. Although more permeable than blood-brain barrier (BBB), this blood-brain tumor barrier (BTB) largely prevents the delivery of non-lipid-permeable chemotherapeutic drugs and monoclonal antibodies to the brain resulting in lack of therapeutic benefit. Primary lung or breast cancers are generally sensitive to therapeutic drugs, but their brain metastases are not. For instance, patients with breast cancer overexpressing HER2/*neu* proto-oncogene of the epidermal growth factor receptor (EGFR) family who were treated with trastuzumab/Herceptin (anti-HER2) have a high rate of brain metastases [7], [8]. It appears to be due to the inability of Herceptin to penetrate BTB to control brain metastases, although it can effectively control primary extracranial breast and lung tumors [9]–[11]. The ability to temporarily increase BTB permeability could dramatically enhance drug delivery to brain tumors, potentially improving the efficacy and reducing side effects. Therefore, understanding the biochemical modulation of BBB and BTB is critical for developing novel safe and effective means of opening barriers for specific drug delivery to brain tumors.

Phosphodiesterase type 5 (PDE5) is a cGMP-specific phosphodiesterase, which selectively inhibits cGMP degradation [12]. PDE5 inhibitors, sildenafil (Viagra®) and vardenafil (Levitra®), are widely used, FDA-approved, oral medicines to treat erectile dysfunction in men [13], [14]. The cyclic guanosine monophosphate (cGMP) is an important intracellular second messenger that has been implicated in the regulation of vascular tone and permeability [15]. Modulation of PDE activity, which can subsequently lead to the intracellular cGMP accumulation, may result in increased permeability of capillaries, including microvessels in brain tumors [16]. Our previous publication showed that orally administered vardenafil and sildenafil not only selectively increased BTB permeability through K_Ca_ channel in gliosarcoma animal models, but enhanced anti-tumor efficacy of a chemotherapeutic agent, doxorubicin [17]. Here we sought to determine whether these marketed PDE5 inhibitors could increase BTB permeability and thereby improve the efficacy of monoclonal antibody treatment of metastatic lung and breast brain tumors positive for HER2/*neu* expression.

## Materials and Methods

### Cell Culture

Mouse brain endothelial cells (MBEC), CRL-5904 cells (human non-small cell lung cancer from a brain metastasis), BT-474 cells (breast cancer cell line) and MDA-MB-435 (breast cancer cell line) were obtained from the American Type Culture Collection (ATCC, Manassas, VA), and were maintained in standard tissue culture conditions. CRL-5904 and BT-474 tumor cell lines are positive for HER2 expression, while MDA-MB-435 has low HER2 expression [18].

### Tumor Implantation

All animal studies were approved by Cedars-Sinai Medical Center Institutional Animal Care and Usage Committee (IACUC) and were conducted in strict accordance to the IACUC protocol #2044.

The metastatic brain tumor xenograft models were established using athymic nude mice (Charles River Laboratories International, Inc., Wilmington, MA). CRL-5904 cells (5×10^4^), BT-474 cells (1×10^5^) or MDA-MB-435 (5×10^4^) in 2 µl of 1.2% methylcellulose/saline were implanted into the striatum respectively with the coordinates of 2.3 mm lateral to bregma and 3.0 mm deep from dura. To establish breast metastatic brain tumor model, estrogen pellets (Innovative Research of America, Sarasota, FL) were implanted subcutaneously in nude mice one week before intracranial tumor implantation.

### *In Vivo* BTB Permeability Study

Fourteen days after tumor implantation, mice were treated with vardenafil (Levitra®, GlaxoSmithKline, Research Triangle Park, NC) at an oral dose of 10 mg/kg for 60 min for the maximal effect [17], bradykinin (1.8 mg/kg; Sigma-Aldrich, St. Louis, MO) for 15 min [19], [20], or saline as control. Mice were then subjected to the regional permeability studies by tail vein injection of radiotracer [^14^C]dextran (molecular weight 70,000 D; 50 µCi/kg; Sigma-Aldrich, St. Louis, MO). The method used to determine the unilateral transport constant *Ki* (µl/g/min), the initial rate for blood-to-brain transfer of radiotracer, has been described in our previous publication with minor modification [17]. In brief, the *Ki* was determined by radiotracer [^14^C]dextran in the tumor core and contralateral brain tissue using quantitative autoradiography (QAR). Quantitative analysis of the regional radioactivity was performed using ImageJ software (National Institutes of Health, Bethesda, MD).

### Herceptin-Alexa Fluor 680 Uptake in Metastatic Brain Tumor Model

Trastuzumab (Herceptin®, Genentech, Inc., San Francisco, CA) was labeled with an Alexa Fluor®680 fluorescent dye (Invitrogen, Carlsbad, CA) using Xenofluor labeling kit (Caliper, Alameda, CA). Tumor-bearing mice were treated with oral vardenafil (10 mg/kg) or saline as control, and then mice were subjected to the permeability studies by tail vein injection of 5 mg/kg Herceptin-Alexa Fluor 680. Xenogen IVIS 200 whole animal fluorescent imager (Caliper Life Sciences, Hopkinton, MA) was used for assessment of drug distribution and localization in isoflurane-anesthetized nude mice at different time points (before drug administration; 1 h, 3 h, 6 h after drug administration). Six hours after drug administration, mice were anesthetized with i.p. ketamine and xylazine and subjected to transcardial perfusion to eliminate the circulating drug in blood vessels. The brains were harvested to detect the fluorescent signal. Signal intensities in the tumor were analyzed by Xenogen Living Image®, Version 2.50 (Caliper Life Sciences, Hopkinton, MA). The brains were snap-frozen in liquid nitrogen and sectioned on a cryostat (Leica Microsystems, Mannheim, Germany). Herceptin-Alexa Fluor 680 accumulation in the tumor cells was further studied by confocal microscopy using Leica confocal laser scanning microscope TCS SP5 (Leica Microsystems, Germany).

### Survival Study in Brain Metastasis Models: Lung and Breast Cancers

For the survival experiments, CRL-5904, BT-474 or MDA-MB-435 tumor-bearing mice were randomly divided into four groups treated as follows: (1) control saline; (2) vardenafil (10 mg/kg, orally, five times per week; dose was based on previous data [17]); (3) Herceptin (10 mg/kg, intravenously via tail vein, twice per week; dose was based on our preliminary data and a previous publication [21]; and (4) Herceptin (10 mg/kg, intravenously, twice per week) plus vardenafil (10 mg/kg, orally, five times per week). Mice received their treatments beginning at day 4 after tumor implantation. CRL-5904 cell implantation and survival experiment was repeated two times with six animals per group. The survival data were very similar and were thus combined together. BT-474 cell line is growing longer and the experiment duration is estimated at about three months. This experiment was performed once with a higher number of animals per group, n = 8. MDA-MB-435 tumor-bearing survival study had 5 to 8 mice per group. Mice were monitored carefully for clinical signs attributable to brain tumor growth or until death. The efficacy of therapy was estimated by the median survival time of the animals.

### Western Blot Analysis of Apoptotic Effect on Brain Tissues of Treated Mice

Normal and tumor mice brain tissues were collected after treatment with saline, vardenafil, Herceptin, and Herceptin plus vardenafil. Samples from five tumors in each group were pooled. Total protein was extracted and concentrations were determined using a BCA assay kit (Bio-Rad Laboratories, Hercules, CA). Equal amounts of protein were loaded on 10% SDS-polyacrylamide gel and transferred to nitrocellulose membranes. For apoptosis detection, the membranes were probed with primary antibodies to cleaved Poly-ADP-Ribose-Polymerase (PARP), an 89-kDa PARP fragment that is considered as a marker of apoptosis, and to an internal control, glyceraldehyde 3-phosphate dehydrogenase (GAPDH; BD Pharmingen, San Diego, CA), respectively. Horseradish peroxidase-conjugated secondary antibodies were used for detection followed by enhanced chemiluminescence development (Bio-Rad Laboratories, Hercules, CA).

### *In Vitro* BBB Permeability Model

To mimic BBB condition *in vitro*, transwell system (Corning, Inc., Corning, NY) was used for cellular drug transport study. MBEC were seeded into the inserts of transwell system at 2×10^4^/well and cultured for 2 to 3 days until confluence. Washed cells were incubated at 37°C for 30 min in culture media without serum as control or in media with 10 µg/ml vardenafil. Then, these media were removed and replaced with [^14^C]dextran at 0.2 µCi/ml in the inserts. After 5 or 30 min incubation, the media from the low chambers were collected and subjected to radioactivity measuring by liquid scintillation counting using a Beckman LS 6000 Scintillation Counter (Beckman Coulter, Fullerton, CA). Triplicate wells were run for each group.

### Drug Uptake by Endothelial Cells

The same numbers of MBEC (2×10^4^/well) were seeded in flat bottom 96-well plates and cultured until confluence. To verify which endocytosis pathways were involved in PDE5 inhibitor-induced drug uptake, several inhibitors were examined by first incubating cultured MBEC for 30 min in serum-free medium as follows: Filipin (1 to 5 µg/ml) and methyl-β-cyclodextrin (2.5 to 10 mM) to inhibit caveolae-mediated endocytosis; amiloride (25 to 50 µm) to inhibit macropinocytosis; chlorpromazine (2.5 to 10 µg/ml) and phenylarsine oxide (7.5 to 30 mM) to inhibit coated pit/clathrin endocytosis pathway [22], [23]. All inhibitors were purchased from Sigma-Aldrich (St. Louis, MO). The medium was replaced with the one containing pathway inhibitors and PDE5 inhibitors (50 µg/ml sildenafil, Viagra®, Pfizer, NY; or 10 µg/ml vardenafil), and cells were incubated at 37°C for 30 min. Then the medium was removed and replaced with Herceptin-Alexa Fluor 680 (25 µg/ml) for 0.5 or 3 hrs. After washing, cells were lysed in 100 µl of 1% (w/v) Triton X-100. Two repeats of this experiment were run, with similar results. Uptake data are expressed as percent of control. Uptake of labeled Herceptin in cultures without endocytosis inhibitors was also tested. A standard curve was obtained in untreated cells using different dilutions of Herceptin-Alexa Fluor 680 in lysis buffer. Fluorescent signals were measured using excitation at 680 nm and emission at 720 nm in a microplate reader (Molecular Devices, Sunnyvale, CA). Triplicate wells were run for each group. Data are expressed as µg Herceptin per one ml of lysis buffer.

### Herceptin-Alexa Fluor 680 Uptake by Cultured MBEC

MBEC were seeded in Lab-Tek™ chamber slides (Thermo Fisher Scientific, Rochester, NY). Washed cells were pretreated with filipin (5 µg/ml) for 30 min in serum-free media followed by incubation at 37°C for 30 min in culture media as control or media with a PDE 5 inhibitor (10 µg/ml vardenafil). Then, this medium was removed and replaced with the one containing Herceptin-Alexa Fluor 680 (25 µg/ml) for 3 hrs. Cells were washed with PBS, fixed with 4% paraformaldehyde for 20 min, and incubated with anti-caveolin-1 (1∶100; BD Biosciences, San Jose, CA) antibodies at 4°C overnight. The signals were detected with Texas Red-conjugated secondary antibodies (1∶200; Jackson ImmunoResearch, West Grove, PA). To detect HER2 expression on cells, they were stained with anti-HER2 (1∶100; Upstate, Temecula, CA) by indirect immunofluorescence. The cells were counterstained with 4′, 6-diamidino-2-phenylindole (DAPI; Vector Laboratories, Burlingame, CA) and cover-slipped. The slides were examined under a Leica confocal microscope. Negative control experiments included omission of primary antibodies and were routinely performed.

### Statistics

Statistical analyses were performed using the Student's *t*-test (Prism4, GraphPad Software, San Diego, CA). Animal survival was analyzed by the Kaplan-Meier method (log-rank test). Data were expressed as mean ± standard error of mean (SEM). p<0.05 was considered significant.

## Results

### Vardenafil Effects on Cell Membrane Permeability *in Vitro* and *in Vivo*

**Figure 1:**
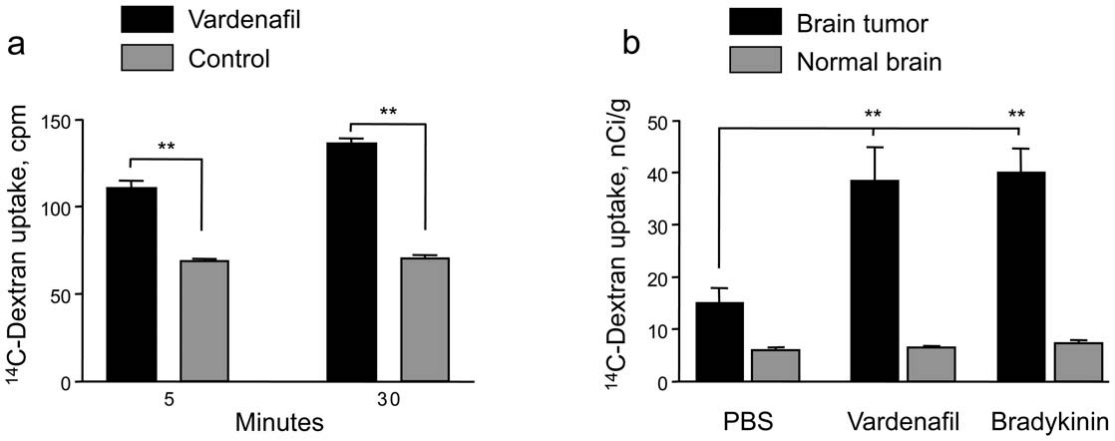
PDE5 inhibitor, vardenafil, increases permeability in an *in vitro* BBB model and in metastatic brain tumors in mice. (a) Vardenafil significantly increases the uptake of [^14^C]dextran (MW 70,000 D) by MBEC compared to medium treated-cells at 5 min (p<0.01) and 30 minutes (p<0.01) respectively in an *in vitro* BBB model. (b) Effects of oral PDE5 inhibitor on the rate of radioactive dextran transport, Ki, into the metastatic brain tumors. Vardenafil (10 mg/kg) was administered orally followed by permeability determination as in a previous publication [17]. Intravenous administration of short-term vascular modulator, bradykinin (1.8 mg/kg) served as a positive control [31], [43]. Oral administration of vardenafil (n = 5) significantly increases *Ki* in the tumor center compared with saline control (n = 5). Intravenous infusion of bradykinin (n = 6) also significantly increases *Ki* in the tumor core compared with control. No permeability increase in the contralateral normal brains. **, p<0.01.

In the *in vitro* setting, a Corning transwell system was used for cellular drug transport study as a model of BBB. Mouse brain endothelial cells were seeded into the transwell inserts for the examination of transcytosis of [^14^C]dextran (MW 70,000 D this molecular weight was selected to compare with big molecular drugs, therapeutic monoclonal antibody in our case). At different intervals after dextran addition, the medium in the low chamber was collected to detect the transported dextran by liquid scintillation counting. The results showed that vardenafil significantly increased the transcytosis of [^14^C]dextran through MBEC compared to medium-treated cells (Figure 1a) both at 5 min (110.67±7.37 vs. 70.67±3.06 cpm; p<0.01) and 30 min (136.33±4.54 vs. 64.00±10.58 cpm; p<0.01).

In the *in vivo* study, vardenafil (n = 5) was given at an oral dose of 10 mg/kg for the maximal effect according to previous publication [17]. Its effects on the initial blood to brain or blood to tumor transport, *Ki*, were studied and compared with that of bradykinin (positive control), which increases BTB transport [17], [24], after infusing with a radiotracer-high molecular weight [^14^C]dextran. Vardenafil treatment resulted in a 2.6-fold increase in BTB permeability (38.49±14.23 nCi/g vs. 15.09±6.09 nCi/g in saline-treated controls; p<0.01). Consistent with previous publications [17], [24], administration of bradykinin (1.8 mg/kg, n = 6) similarly increased the *Ki* in the brain tumors (40.06±11.13 vs. 15.09±6.09 nCi/g; p<0.01) as compared to the saline-treated controls (n = 5). Transport of [^14^C]dextran in normal brain was not affected by the treatments (Figure 1b).

**Figure 2:**
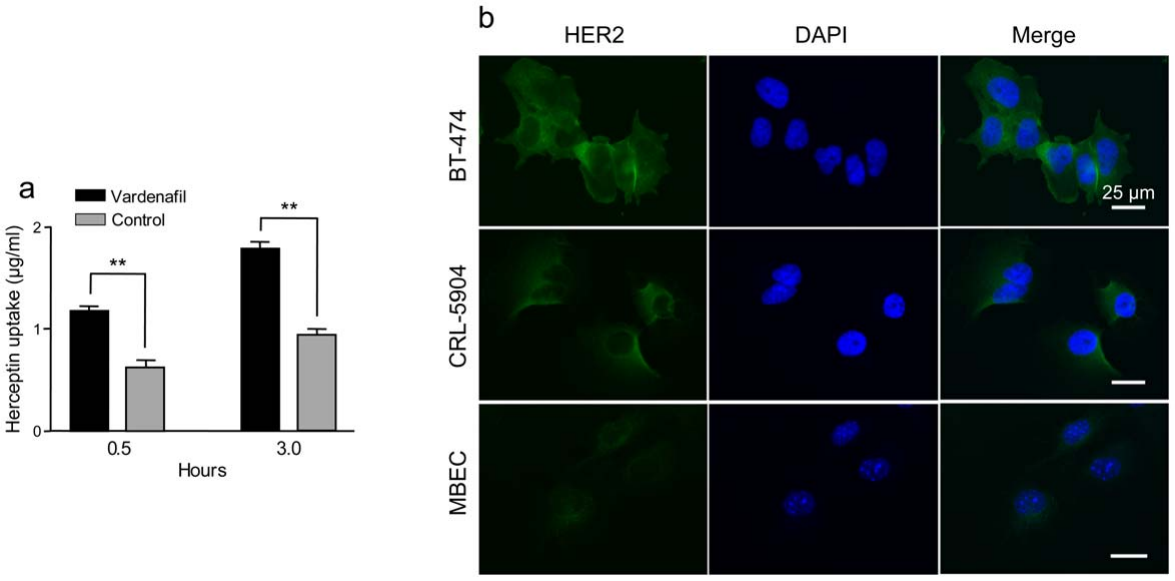
PDE5 inhibitor induces permeability increase of therapeutic antibody, Herceptin. (a) PDE5 inhibitor vardenafil significantly increases Herceptin-Alexa Fluor 680 uptake by cultured MBEC compared to medium treated-cells at 0.5 and 3 hrs (close to 2-fold; vardenafil vs. control). **, p<0.01. (b) HER2 expression on different cell lines. BT-474 breast cells have the highest HER2 expression, followed by CRL-5904 cells. HER2 expression on MBEC is not detected. Lack of HER2 on MBEC suggests that Herceptin could not be internalized by receptor-mediated clathrin-dependent endocytosis and may account for absence of effect observed with the corresponding inhibitors, chlorpromazine and phenylarsine oxide (Figure 4). Nuclei are counterstained with DAPI. Scale bar  = 25 µm.

To examine *in vitro* uptake of Herceptin-Alexa Fluor 680, endothelial cells were incubated with labeled drug with or without preincubation with vardenafil. Vardenafil-treated MBEC showed significantly increased Herceptin-Alexa Fluor 680 uptake (Figure 2a) compared to medium only control (1.19±0.03 vs. 0.65±0.07 µg/ml, p<0.01 at 30 min; 1.79±0.07 vs. 0.95±0.05 µg/ml, p<0.01 at 3 hrs). It should be noted that MBEC did not express surface HER2 in contrast to CRL-5904 and BT-474 HER2-postive tumor cell lines used (Figure 2b).

### Mechanisms of Antibody Delivery Through Endothelial System *in Vitro*

**Figure 3:**
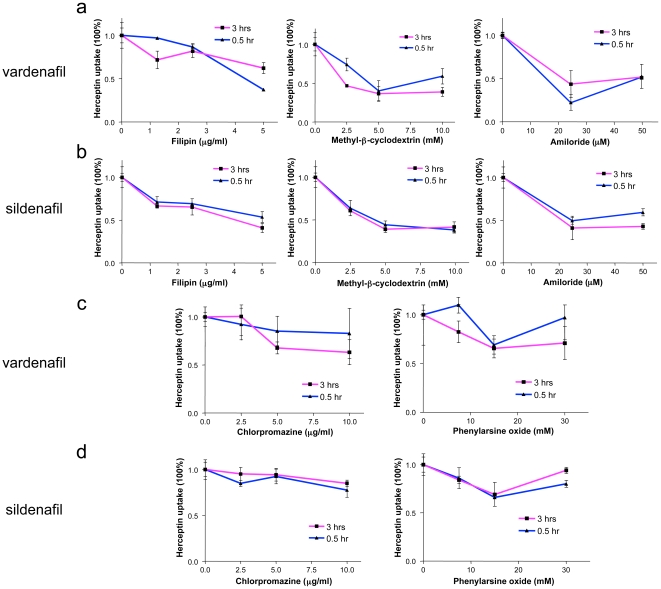
Examination of the involvement of major endocytic pathways in PDE5 inhibitors-induced permeability increase. Each inhibitor was tested in different dosages and at two incubation time points (0.5 and 3 hrs). (a, b; left graphs) Filipin, a caveolae pathway inhibitor, suppresses the effect of vardenafil (50.1±12.4% inhibition) and sildenafil (52.7±6.3% inhibition) on cultured MBEC at different concentrations (1.25, 2.5, and 5 µg/ml). (a, b; middle graphs) Methyl-β-cyclodextrin, another caveolae pathway inhibitor, has the same concentration-dependent (2.5 to 10 mM) inhibition on vardenafil (51.1±10.0% inhibition) and sildenafil (60.0±1.7% inhibition) effects. (a, b; right graphs) Amiloride, a macropinocytosis inhibitor, blocks the effect of vardenafil (58.8±3.6% inhibition) and sildenafil (49.1±8.2% inhibition) at concentrations of 25 and 50 µM. (c, d; left graphs) Chlorpromazine (2.5, 5.0, and 10 µg/ml), a clathrin pathway inhibitor, does not significantly reduce the effect of vardenafil (27.0±9.8% inhibition) or sildenafil (18.8±3.8% inhibition) on cultured MBEC. (c, d; right graphs) Phenylarsine oxide (7.5, 15, and 30 mM), another clathrin pathway inhibitor, equally doesn't block the effect of vardenafil (16.0±13.1% inhibition) or sildenafil (12.9±7.0% inhibition) on cultured cells. Data in parentheses are expressed as inhibition at high dose (mean ± SEM).

**Figure 4:**
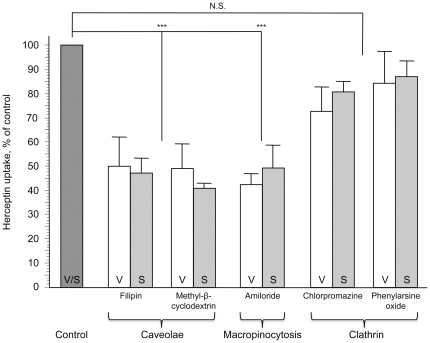
Effect of inhibitors of three major endocytic pathways on reversing vardenafil and sildenafil enhancement of Herceptin uptake. Bar graph represents high dosage results in Fig. 3, averaged for both 0.5 and 3 hrs of incubation (mean ± SEM). Control, Herceptin uptake by cells incubated with PDE5 inhibitors (data on vardenafil and sildenafil were similar, so they were combined and taken as 100%). Both inhibitors of caveolae endocytic pathway (filipin and methyl-β-cyclodextrin) significantly reduced PDE5 inhibitor effect compared to vardenafil/sildenafil-stimulated control, bringing Herceptin uptake to baseline level. The same was true for macropinocytosis inhibitor, amiloride. The effect of either inhibitor of coated pit/clathrin pathway (chlorpromazine or phenylarsine oxide) on Herceptin uptake was non-significant (N.S.). V, vardenafil; S, sildenafil; MPC, macropinocytosis. ***, p<0.001.

To explore the underlying mechanisms of PDE5 inhibitors-induced BTB permeability increase, we have examined the role of three major pathways for substance endocytosis, such as clathrin- and caveolae-mediated transport, as well as macropinocytosis. We further investigated whether increased transport induced by PDE5 inhibitors was associated with any of these major mechanisms (Figures 3, 4). Caveolae-mediated endocytosis was inhibited by filipin and methyl-β-cyclodextrin [22], [23]. The vardenafil- or sildenafil- enhanced uptake of Herceptin-Alexa Fluor 680 was inhibited by 50-60% by the pretreatment of the cells with filipin or methyl-β-cyclodextrin (Figure 3 a, b; left and middle graphs; Figure 4, p<0.001 vs. vardenafil/sildenafil-stimulated control). Because vardenafil and sildenafil increased Herceptin uptake *in vitro* about two-fold (Figure 2a), caveolae pathway inhibitors agents essentially brought Herceptin uptake down to control levels without PDE5 inhibitors. Next, we examined the role of macropinocytosis by using its inhibitor, amiloride [22], [25]. The uptake of Herceptin-Alexa Fluor 680 increased by vardenafil or sildenafil was also similarly inhibited by amiloride pretreatment (Figure 3 a, b; right graphs; Figure 4; p<0.001 vs. vardenafil/sildenafil-stimulated control). However, clathrin pathway inhibitors, chlorpromazine and phenylarsine oxide, did not significantly interfere with the effects of vardenafil or sildenafil (Figure 3 c, d; Figure 4; p>0.05 vs. vardenafil/sildenafil-stimulated control), possibly due to lack of HER2 receptor expression on MBEC (Figure 2b). Therefore, stimulation of caveolae and macropinocytosis pathways may play a major role in PDE5 inhibitor effect on Herceptin uptake.

**Figure 5:**
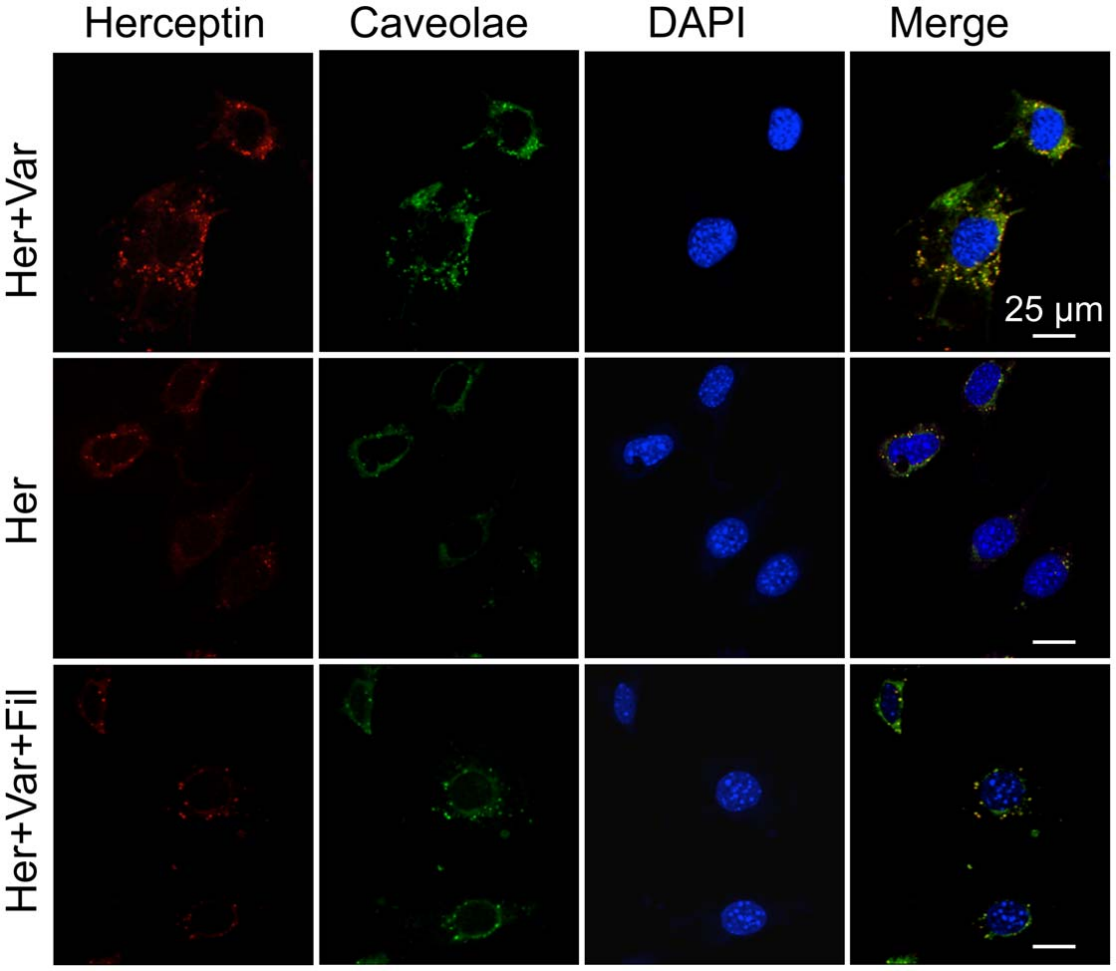
Co-localization of Herceptin-Alexa Fluor 680 with caveolae in cultured MBEC. Cultured cells have low uptake of Herceptin (Her), which is markedly increased when co-administered with vardenafil (Var). Note pronounced co-localization of Herceptin with caveolin-1. The enhancing effect of vardenafil is virtually abolished by filipin (Fil). Vardenafil was used at 10 µg/ml. Nuclei were counterstained with DAPI. Scale bar  = 25 µm.

To further examine the effect of PDE5 inhibitors on drug uptake, we detected Herceptin-Alexa Fluor 680 in cultured MBEC using confocal microscopy. Caveolae expression was monitored by immunocytochemical co-staining with an antibody to caveolin-1. With vardenafil pretreatment, Herceptin-Alexa Fluor 680 uptake by MBEC was higher compared with Herceptin alone and filipin-pretreated cells; the latter two were similar to each other (Figure 5). Filipin (Figure 5), methyl-β-cyclodextrin, and amiloride (data not shown here) all blocked the PDE5 inhibitor-induced increase in Herceptin uptake, suggesting that the effects of PDE5 inhibitors are due to the activation of caveolae-mediated endocytosis or macropinocytosis.

### Effect of PDE5 Inhibitors on Herceptin Uptake by Metastatic Brain Tumors

**Figure 6:**
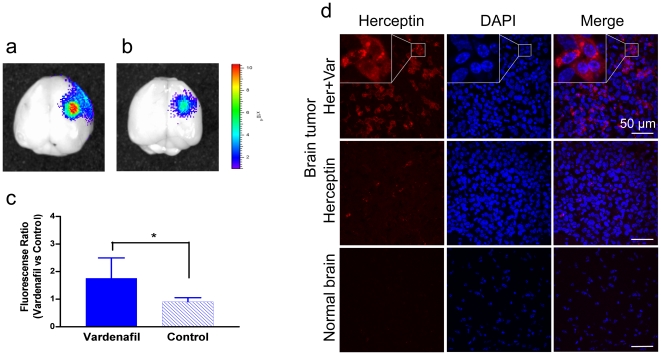
Effects of vardenafil on the rate of Herceptin transport and accumulation in metastatic brain tumor CRL-5904 bearing animal model. Vardenafil was administered orally at 10 mg/kg followed by tail vein injection of Herceptin-Alexa Fluor 680. (a) Brain tumors treated with Herceptin and vardenafil; (b) Brain tumors treated with Herceptin and saline; fluorescence signals were detected by Xenogen IVIS 200 imaging. (c) Quantitation of Herceptin accumulation in the tumor. Oral administration of vardenafil (n = 5) significantly increased Herceptin uptake by the brain tumors compared with saline control (n = 5). * p<0.05. (d). Herceptin uptake and accumulation in the metastatic brain tumor tissue. Sections of a tumor after Herceptin plus vardenafil treatment have markedly brighter fluorescence with significantly more positive cells compared with saline-treated control. No fluorescence signal was detected in the contralateral normal brain tissue. Nuclei were counterstained with DAPI. Scale bar  = 50 µm.

The *in vitro* data prompted us to test whether PDE5 inhibition could also increase therapeutic antibody uptake by the metastatic brain-implanted tumors. We chose Herceptin because it is a FDA-approved humanized monoclonal antibody to treat HER2-positive tumors. It also has limited access across BBB/BTB [9], [26]. Herceptin was labeled with Alexa Fluor 680 fluorescent dye for detection by Xenogen IVIS 200 imaging system. Oral administration of vardenafil (10 mg/kg, n = 5) resulted in significant 2-fold increase of Herceptin-Alexa Fluor 680 transport in brain tumor compared with saline treated-mice (Figure 6 a-c; n = 5; p<0.05). Herceptin-Alexa Fluor 680 signal was not detected in the contralateral normal brains (Figure 6a, b). The accumulation of Herceptin was further observed on brain tumor cryostat sections by confocal microscopy. Sections of vardenafil-treated tumors had markedly higher drug accumulation compared with Herceptin alone (Figure 6d) or saline-treated controls. No fluorescence signal was detected in the contralateral normal brain tissue.

### Treatment of Lung and Breast Intracranial Tumors

**Figure 7:**
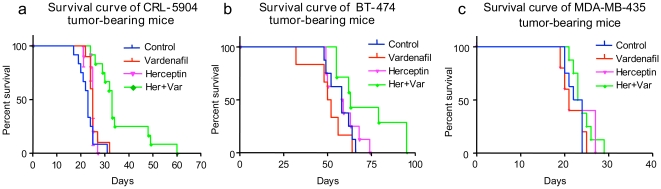
Effect of vardenafil on Herceptin therapy in metastatic brain tumor-bearing mice. (a) Effect of vardenafil on the survival rates of CRL-5904 metastatic brain tumor-bearing mice. Groups of mice (control, n = 12; Herceptin, n = 10; vardenafil, n = 10; Herceptin + vardenafil, n = 12) were treated with saline, Herceptin (10 mg/kg, i.v.), vardenafil (10 mg/kg, oral), or Herceptin (10 mg/kg, i.v.) plus vardenafil (10 mg/kg, oral). All mice were treated starting on the fourth day after tumor implantation. The Kaplan–Meier survival curves showed that mice treated with Herceptin plus vardenafil survived significantly longer than any other of the three groups of mice (p<0.01). (b) Effect of vardenafil on Herceptin therapy in BT-474 (breast cancer) metastatic brain tumor-bearing mice. Groups of mice (control, n = 8; vardenafil, n = 6; Herceptin, n = 8; Herceptin + vardenafil, n = 7) were treated with saline, Herceptin, vardenafil, or Herceptin plus vardenafil in the same way as CRL-5904 tumor-bearing animals. The Kaplan–Meier survival curves again showed that mice treated with Herceptin plus vardenafil survived significantly (p<0.02) longer than any other of three groups of mice. (c) Effect of vardenafil on Herceptin therapy in MDA-MB-435 (breast cancer with low HER2 expression) metastatic brain tumor-bearing mice. Groups of mice (control, n = 8; vardenafil, n = 5; Herceptin, n = 5; Herceptin + vardenafil, n = 8) were treated with saline, vardenafil, Herceptin, or Herceptin plus vardenafil in the same way as mentioned above. The Kaplan–Meier survival curves showed that there was no significant difference among the treatment groups (p>0.05).

To determine if the permeability increase in metastatic brain tumors by the PDE5 inhibitors can be translated into improved efficacy of tumor therapy, Herceptin was administered to mice with brain-implanted lung metastatic CRL-5904 and breast cancer BT-474 tumors positive for HER2/*neu*. CRL-5904 bearing mice were treated with Herceptin (10 mg/kg, twice per week, intravenously) [21] beginning at day 4 after tumor implantation, with oral administration of vardenafil (10 mg/kg, 5 times per week). Log-rank analysis of the Kaplan–Meier survival curves showed a significant increase (p<0.01) in survival of mice treated with Herceptin plus vardenafil as compared to the saline-treated, vardenafil alone- or Herceptin alone-treated mice (Figure 7a). The mean survival time for mice treated with Herceptin plus vardenafil combination increased by about 30% and was significantly longer (35±8 days) compared to those of saline- (22±2 days), Herceptin alone- (25±2 days), and vardenafil alone-treated mice (25±2 days). In mice bearing intracranial BT-474 breast tumors, log-rank analysis of the Kaplan–Meier survival curves also showed a significant survival increase (p<0.02) in the group treated with Herceptin plus vardenafil as compared to the saline-treated, Herceptin alone- or vardenafil alone-treated mice (Figure 7b). The mean survival time for mice treated with Herceptin plus vardenafil was significantly longer (72±18 days) compared to those of saline- (57±7 days), Herceptin alone- (59±9 days), and vardenafil alone-treated mice (50±11 days). Survival with Herceptin plus vardenafil was thus about 20% longer than with Herceptin alone. However, there was no beneficial effect of vardenafil on Herceptin treatment of mice bearing low HER2 expressing MDA-MB-435 tumor (p>0.05; Figure 7c).

**Figure 8:**
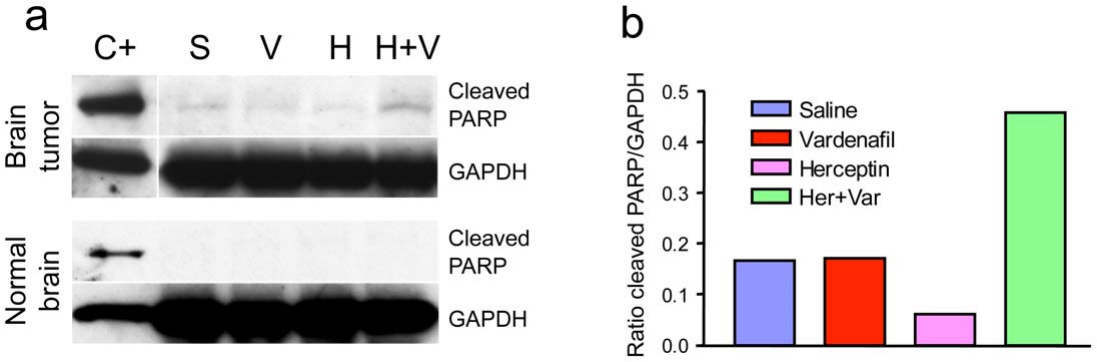
Effect of treatments on cell apoptosis in the CRL-5904 brain tumors. (a) Western blot analysis of an apoptotic maker, cleaved PARP, is shown. The cleaved PARP band has higher expression in the brain tumor lysates (brain tumor) after treatment of mice with Herceptin plus vardenafil (H+V) compared to Herceptin alone (H), vardenafil alone (V) or saline (S). Lane C+, positive controls of cleaved PARP and GAPDH (internal housekeeping control). No cleaved PARP band is observed in the contralateral normal brains in any treatment group (normal brain). (b) The ratio of cleaved PARP versus GAPDH in CRL-5904 brain tumors was plotted using ImageJ. Brain tumors after co-treatment with Herceptin and vardenafil have increased apoptosis as compared to other treatments.

Herceptin binds to the extracellular domain of HER2 and inhibits proliferation and survival of HER2-dependent tumors by promoting DNA fragmentation associated with apoptotic cell death [27]. In addition to monitoring survival, we also detected the apoptotic effect on the brain tissue after the treatments. The brain tumor tissues and contralateral normal brain tissues were collected, pooled at five per group, and subjected to apoptotic assay by Western blot for cleaved 89-kDa fragment of poly-ADP-ribose-polymerase (PARP) as a marker of apoptosis. CRL-5904 brain tumors treated by Herceptin plus vardenafil showed about two-fold higher level of cleaved PARP compared with untreated, vardenafil alone- or Herceptin alone-treated brain tumors (Figure 8a, top). The ratio of cleaved PARP relative to housekeeping protein GAPDH showed marked increase in the Herceptin plus vardenafil group compared to all other groups (Figure 8b). We did not detect any cleaved PARP in the contralateral normal brain tissues with different treatments (Figure 8a, bottom). The enhanced apoptosis in the Herceptin plus vardenafil treated brain tumor tissue indicated that vardenafil increased uptake and efficacy of Herceptin in the brain tumors. In BT-474 tumors we did not detect a significant increase in cleaved PARP in vardenafil plus Herceptin treatment compared with other groups, maybe due to the longer survival time (up to 3 months) in this animal model.

## Discussion

In this study, we sought to determine whether cGMP-specific PDE5 inhibitors could increase the rate of delivery of large macromolecular drugs exemplified by therapeutic antibodies across the BTB in order to improve efficacy of drug therapy for metastatic brain tumors. We have also examined possible mechanisms underlying PDE5 inhibitor action, related to changes in vesicular transport. It is shown here that oral administration of PDE5 inhibitor vardenafil selectively increased the rate of brain tumor accumulation of intravenously administered [^14^C] high molecular weight dextran and a monoclonal antibody trastuzumab/Herceptin against HER/*neu*. Importantly, Herceptin in combination with vardenafil significantly improved the survival of metastatic brain tumor-bearing mice with increased tumor apoptosis. Inhibitors of caveolae endocytosis and macropinocytosis pathway could block PDE5 inhibitor-induced Herceptin uptake by cultured mouse brain endothelial cells, suggesting that stimulation of these pathways was a likely mechanism of PDE5 inhibitor action. Collectively, these findings suggest that PDE5 may serve as an effective target for pharmacological modulation of BTB permeability to enhance delivery and therapeutic effect of otherwise inefficient drugs in the metastatic brain tumors.

Vardenafil and sildenafil are oral drugs that are currently used to treat erectile dysfunction in men. They selectively inhibit the activity of cGMP-specific PDE5, and subsequently increase the cGMP level [12]. Our previous study showed that oral administration of vardenafil and sildenafil selectively increased BTB permeability (1.8-fold and 2.7-fold, respectively) compared with control groups. Transport across the BTB into tumor tissues reached the maximum at 60 to 75 min after administration of sildenafil and vardenafil [17]. In the present study, we chose the optimal dose of vardenafil at 10 mg/kg to treat metastatic brain tumor-bearing mice. Orally administered vardenafil selectively increased permeability of metastatic brain tumors, but not the contralateral normal brain to high molecular weight [^14^C] dextran. Bradykinin that is known to transiently open up BTB was included in this study as a positive control. As expected, bradykinin resulted in a significant increase in BTB permeability, which is consistent with our previous observations [16], [28], [29]. However, bradykinin has a short half-life [30], and its effect on transport into tumors is diminished within 15–20 min after infusion [24], [31], which makes the clinical use of bradykinin difficult. Vardenafil was found to be not only as effective as bradykinin in increasing drug transport into brain tumors, but to also extend the BTB opening up to 105 min, which would give a serious advantage for delivering therapeutics into brain tumors in clinic [17].

Two major cellular mechanisms have been suggested to account for increased BTB permeability: vesicular transcellular transport and tight junction-controlled paracellular permeability of endothelial cells [32]–[34]. Our previous studies indicated that increased vesicular transport was an important mechanism for enhanced drug delivery via biochemical modulation of BTB [19], [34], [35]. We also showed that vardenafil treatment did not significantly affect tight junction integrity in tumor capillary endothelium [17]. These data suggested that tight junction-controlled paracellular mechanism might not play a key role in the BTB permeability increase by vardenafil. Therefore, we have examined the role of three major pathways for substance endocytosis, including coated pit/clathrin- and caveolae-mediated transport, as well as macropinocytosis, as possible mediators of vardenafil action. Our data demonstrate that inhibitors of caveolae or macropinocytosis could abolish PDE5 inhibitor-induced Herceptin-AlexaFluor-680 uptake in cultured MBEC, suggesting the involvement of these pathways in vardenafil effects. Although many molecules are preferentially internalized by one major endocytic pathway [36], it is not uncommon for certain molecules to use several endocytic pathways at the same time [37]. Moreover, PDE5 inhibitors enhance BTB permeability through increasing the cGMP level [12], [17]. It was also reported that the vascular smooth muscle relaxation induced by cGMP was impaired in the presence of methyl-β-cyclodextrin, a caveolae pathway inhibitor [38], further supporting the role of caveolae pathway in PDE5 inhibitor-induced BTB permeability increase. Caveolae are most abundant in continuous capillary endothelia [39] and could be a suitable means for the transfer of anti-cancer drugs or other therapeutics from blood to brain [40]. Receptor (clathrin)-mediated endocytosis mechanism of Herceptin delivery did not appear to play an important role, possibly due to lack of HER2 receptor expression on MBEC.

Herceptin is a humanized monoclonal antibody for treatment of patients with primary breast, non-small lung or prostate cancers that overexpress HER2. Herceptin minimally crosses the BBB/BTB [9], which may be a reason for lack of Herceptin efficiency in treating tumor metastases to the brain/central nervous system [2], [6], [41]. In this study, we showed that oral administration of vardenafil increased Herceptin delivery to metastatic brain tumors through BTB, but not through BBB to contralateral brain tissue. This resulted in a specific accumulation of the antibody in the brain tumor but not in the normal brain, apparently leading to enhanced anti-tumor effect of Herceptin and increased survival of treated mice with HER2-positive lung and breast cancers implanted into the brain as a model of metastasis. This beneficial effect on animal survival was clearly related to Herceptin action because it was not observed in low HER2 expressing tumors. The HER2-positive brain tumors treated with Herceptin plus vardenafil showed significantly higher level of cleaved PARP (apoptotic marker) compared with other treatment groups, consistent with known proapoptotic mechanism of Herceptin action [27].

Taken together, the presented data demonstrate that oral administration of PDE5 inhibitors selectively increases transport across brain tumor capillaries and significantly enhances the anti-tumor effect of Herceptin in mouse models of metastatic HER2/*neu*-positive brain tumors. The results suggest that PDE5 inhibitors may exert their effect through stimulating caveolae-mediated endocytosis and macropinocytosis.

Because lung and breast cancers are the most common primary tumors for brain metastases, treating brain metastases with clinically feasible strategies may have an important effect on disease outcome. Our data suggest that, for metastatic brain tumors positive for EGF family receptors, such as HER2 or EGFR, PDE5 inhibitors may offer a possibility of efficacious treatment using pertinent antibodies (trastuzumab and cetuximab, respectively) that are otherwise inefficient. Potentially, they could also be applied together with CD20 antibody rituximab to treat brain metastases of lymphoma. Because PDE5 inhibitors seem to increase BTB permeability to high molecular weight drugs, they might enhance brain tumor accumulation and thus, therapeutic efficacy of other classes of drugs including emerging nanomedicines [42]. This strategy to circumvent the BBB/BTB may significantly expand the treatment options for patients with brain metastases.
